# Caucasian and south Asian men show equivalent improvements in surrogate biomarkers of cardiovascular and metabolic health following 6-weeks of supervised resistance training

**DOI:** 10.12688/f1000research.15376.2

**Published:** 2019-03-05

**Authors:** Allan Knox, Nicholas Sculthorpe, Fergal Grace

**Affiliations:** 1 Exercise Science Department, California Lutheran University, Thousand Oaks, CA, 91360, USA; 2 Institute of Clinical Exercise and Health Science, University of the West of Scotland, Hamilton, South Lanarkshire, ML3 0JB, UK; 3 School of Health Science and Psychology, Federation University, Ballarat, Victoria, 3350, Australia

**Keywords:** Resistance, Strength, Exercise, Training, South, Asian, Cardiovascular, Metabolic

## Abstract

**Background**: The South Asian population have greater cardiovascular risk than their age-matched Caucasian counterparts, characterized by unfavorable biomarkers. South Asians may also be partially resistant to the pleiotropic benefits of physical activity on cardiovascular health. There is a current absence of studies that compare markers of cardio-metabolic health between Caucasians and South Asians employing resistance exercise. This study set out to compare the response in biomarkers of cardio-metabolic health in Caucasians and South Asians in response to resistance exercise.

**Methods**: Caucasian (n=15, 25.5 ± 4.8 yrs) and South Asian (n=13, 25.4 ± 7.0 yrs) males completed a 6-week progressive resistance exercise protocol. Fasting blood glucose, insulin, and their product insulin resistance (HOMA-IR), triglycerides (TRIGS), low density lipoprotein (LDL), high density lipoprotein (HDL), total cholesterol (TC), vascular endothelial growth factor (VEGF), asymmetric dimythylarginine (ADMA), L-arginine (L-ARG) and C-reactive protein (CRP) were established at baseline and following resistance exercise.

**Results**: There were significant improvements in fasting glucose, TC, LDL, HDL and VEGF in both groups following resistance exercise ( *p*<0.05, for all). No change was observed in insulin, HOMA-IR, TRIGS, ADMA, L-ARG following resistance exercise ( *p*>0.05, in both groups). CRP increased in the South Asian group ( *p*<0.05) but not the Caucasian group ( *p*>0.05)

**Conclusions**: The cardio-metabolic response to resistance exercise is comparable in young Caucasian and South Asian males though inflammatory response to exercise may be prolonged in South Asians.

## Introduction

Diseases that centre on the cardiovascular (CVD) and glycolytic systems are primary contributants to annual global mortality, accounting for approximately 17.7 million and 1.6 million deaths in 2015, respectively ( WHO, 2017a; WHO, 2017b). However, there is considerable heterogeneity of CVD prevalence across racial groups, where the immigrant South Asian (SA) community of the United Kingdom have an approximate 50–100% elevation in cardio-metabolic risk compared with the general population ( Wild *et al*., 2007). This translates to an estimated 5.3 year earlier occurrence of CVD in SAs when compared with their Caucasian (CAUC) counterparts ( Hughes *et al*., 1989).

Pronounced CVD risk amongst SAs is due in part, to the presence of consistently augmented traditional risk factors. For instance, unfavourable lipid profiles been reported in SAs in comparison to CAUCs ( Misra & Khurana, 2011), due to inherent variation of lipid particles. SAs exhibit smaller low density lipoprotein (LDL) particles and dysfunctional high density lipoprotein (HDL) particles compared with CAUCs ( Dodani, 2008; Tziomalos *et al*., 2008). Premature development of Type 2 diabetes (and associated complications) occur approximately 10 years earlier in SAs than CAUCs, and data from large-scale studies such as United Kingdom Asian Diabetes Study (UKADS) reported diabetes related deaths in SAs occurring 7.4 years earlier than CAUCs ( Bellary *et al*., 2010). Comparably higher systemic C-reactive protein (CRP) with regional obesity of the abdomen is another aspect of cardio-metabolic disease that contrives to increase CVD in SAs when compared with CAUCs. The latter has required revisions in the ‘normal’ BMI thresholds for overweight and obese SAs ( Gray *et al*., 2011). These racially specific reclassifications are based on data demonstrating that comparable levels of glycaemia and lipid profiles are seen at BMI levels of 21–26 kg/m ^2^ in SAs compared to 30 kg/m ^2^ in CAUCs ( Gray *et al*., 2011).

Physical exercise is a well-known prophylactic for the development of CVD. In this respect, the cardio protective effects of aerobic exercise are well documented in comparison to fewer studies of resistance exercise (RES). RES has been shown to promote healthogenic effects by improving lipid profiles ( Kelley & Kelley, 2009; Sheikholeslami Vatani *et al*., 2011), insulin sensitivity ( Mann *et al*., 2014; Schwingshackl *et al*., 2014), and CRP levels ( Donges *et al*., 2010). However, these studies almost exclusively enrol CAUC participants with the result that there is a general paucity of RES studies and resultant data amongst the SA population.

Few investigations have included SA participants and have used lifestyle modification methodologies which have produced modest results. For instance, a 3-year lifestyle intervention, promoting beneficial dietary and physical activity behaviours in SAs report trivial effects in body mass and no change in either blood pressure or fasting blood glucose ( Bhopal *et al*., 2014). Other investigations that have studied the interaction between physical activity and cardio-metabolic risk in SAs have also found encouraging results. Data from the Health Survey of England show an inverse association between the participation of 30 minutes of moderate intensity exercise per week and cardio-metabolic risk in CAUCs and SAs ( Williams *et al*., 2011). However, findings from the Indian Diabetes Prevention Programme and the ADDITION-Leicester study identified a blunted response in cardio-metabolic biomarkers to physical activity in the SA cohort compared with CAUCs ( Yates *et al*., 2010), which corroborates the findings from other racial comparison studies ( Fretts *et al*., 2009; Steinbrecher *et al*., 2012). Recently, IIiodromiti and colleagues (2016) have made a valuable addition to the literature by offering that SAs would require ~50% greater participation in physical activity than CAUCs (232 min/week ^-1^ vs 150 min/week ^-1^ in CAUCs) to address the imbalance in healthogenenic benefits of aerobic exercise between these racial groups.

While these data may have potentially important consequences for exercise prescription for SAs, the field is limited by the dearth of objective assessment of exercise participation. Our previous work has demonstrated differences in muscular strength adaptation in response to supervised RES in SAs, which may translate to an attenuated cardio-metabolic response ( Knox *et al*., 2017). These findings are in line with two previous studies which compared objectively determined physical activity ( Iliodromiti *et al*., 2016) and aerobic exercise ( Hall *et al*., 2010) which support the potential for a blunted response to physical activity in SAs compared with CAUCs. However, no study has explored this phenomenon using the medium RES. Therefore, the purpose of this study was to assess whether the different response of exercise in the SA population suggested by previous reports is applicable to RES. We will address this aim by establishing the response of traditional cardio-metabolic biomarkers to 6-weeks progressive RES between young, healthy CAUC and SA males. We hypothesised that 1) differences in biomarkers of cardio-metabolic health are evident at baseline (PRE) between CAUCs and SAs, 2) differences in biochemical markers in both groups following RES (POST), 3) both groups differ at POST in any biochemical marker of cardio-metabolic health.

## Methods

### Participants

This study was carried out in accordance with the recommendations of the University of the West of Scotland. The protocol was approved by the School of Science and Sport ethics committee (approval: HREC_Sci2013/02/Knox). All subjects gave written informed consent in accordance with the Declaration of Helsinki. In the present study, the main outcome measure was squat performance. All participants were recruited by local advertisement and word of mouth. Inclusion criteria were that (i) participants did not engage in any recreational or competitive sports, (ii) were naive to RES prior to study enrolment, (iii) no history of cardio-metabolic disease prior to study participation (iv) or any illness that may provide a contraindication to exercise participation. Only male participants were recruited to control for the effect of sex on potential responses. Race and familial generation of United Kingdom (UK) patriotage was self-reported.

### Anthropometrics

All anthropometric measurements were performed by AK in the Human Performance Laboratory of the University of the West of Scotland (HPLUWS). Height was determined using a portable stadiometer (Leicester Height Measure, Seca, Birmingham, U.K.). Body mass was measured to the nearest 0.1 kg using commercially available scales (TBF-300, Tanita, Tokyo, Japan), where participants were required to remove footwear and unnecessary clothing before measurement which were obtained in adherence with manufacturers guidelines. Body mass index was calculated using the following formula; BMI = body mass (kg) ÷ height (m) ^2^. Total body fat percentage (%) was calculated using bioelectrical impedance analysis (BIA) using a commercially available analyser (body composition analyser TBF-300, Tanita, Tokyo, Japan). Criterion validity of this method has been suitably comparable (r = 0.952) compared with the gold standard dual energy x-ray absorptiometry (DEXA) ( Bozkirli *et al*., 2007).

Waist circumference (WC) and waist-hip ratio (WHR) was calculated according to the guideline published by the WHO ( WHO, 2008) using a commercially available ergonomic circumference measuring tape (Seca 201, Seca, Birmingham). Participants were asked to remove any clothing on their upper body before standing with their arms by their side with their feet positioned close together with their body weight distributed evenly across both feet. For WC, the tape was placed around the approximate midpoint between the lower palpable rib and the top of the iliac crest ( WHO, 2008). The participant was then asked to relax and take several deep breaths to account for any abdominal tension caused by the nature of this procedure where criterion measurement was taken at the end of normal expiration to control for any diaphragm movements. Hip circumference (HC) was calculated by measuring the circumference of the widest portion of the buttocks.

### Systemic blood pressure

Systemic blood pressure data was collected by AK in the HPLUWS. Arterial blood pressure (BP) was measured by an automated BP monitor (M6, Omron, Milton Keynes, U.K.). Participants sat in a quiet room for 10 minutes with their legs uncrossed and with the arm being used for measurement supported approximately level with the heart. A total of 3 readings of systolic blood pressure (SBP) and diastolic blood pressure (DBP) were recorded. The final data was calculated from the mean values of the three reading. From these values, it was possible to calculate mean arterial pressure (MAP) the following formula; MAP = 0.33(SBP-DBP) + DBP.

### Blood biochemistry

All blood sampling and analysis was performed by AK in the HPLUWS. To account for diurnal variation in blood variables, participant blood was sampled at the same time of day and following a 12-hour fast. Briefly, each participant remained in the supine position for at least 30 minutes during sample collection enabling control of plasma volume shifts ( Hagan *et al*., 1978). Venous blood was collected from the antecubital vein via venepuncture. Blood glucose, triglycerides (TRIGS), HDL and total cholesterol (TC) were determined by spectrophotometery (Rx Monza, Randox Laboratories, UK). Samples were mixed with respective reagents and incubated at room temperature for the duration stated by reagent manufacturer (Randox Laboratories, UK) before being inserted into the spectrophotometer and read at the corresponding wavelength. LDL concentrations were established indirectly by the following Friedwalde equation ( Friedewald *et al*., 1972); LDL = TC - HDL - (TRIGS/5). This method was appropriate as all samples did not exceed fasting TRIGS concentrations of 4.52 mmol/L and were free from chylomicrons and hyperlipidaemia. Insulin (Alpco Diagnostics Salem, USA: catalogue number: 80-INSHU-E01.1), vascular endothelial growth factor (VEGF: Invitrogen, Life Technologies, USA: catalogue number: BMS277-2), asymmetric dimythylarginine (ADMA) and L-arginine (Diagnostika GMBH, Hamburg, Germany: catalogue number: EA207/192) concentrations were established using commercially available ELISA kits. CRP was determined by commercially available immunoturbidimetric assay (Rx Monza, Randox laboratories, UK: catalogue number: CP3885). Insulin resistance was calculated from the standard formula: (fasting glucose × fasting insulin)/22.5. A small subsample (n=6 from both groups) was used to establish changes in ADMA and L-arginine for hypothesis generating purposes.

### Muscular strength and resistance exercise protocol

Muscular strength assessments were performed by AK in the HPLUWS. All training sessions were performed in local authority fitness centres under the supervision of AK. Measurement of muscular strength, prescribed training protocol, and associated data has been previously reported ( Knox *et al*., 2017). Briefly, lower and upper body muscular strength was assessed by the 3 repetition maximum protocol utilizing the squat and bench press exercise, respectively. The protocol utilised 5 main compound exercises including back squats, bench press, deadlifts, shoulder press, and lateral pull down and progressed in a linear approach three times per week. Each exercise was prescribed at 3 sets of 10 repetitions with 2 minutes’ rest between sets. The training programme was separated into two sessions; Session A and Session B, with each being performed consecutively throughout the duration of the study. Training days were separated by at least one day but no longer than two days.

### Statistical analysis

Data were analysed using SPSS version 24.0 for Windows. A power calculation was performed using G*Power V3 with reference to previously published data regarding squat strength in healthy, untrained, young men ( MacDonald *et al*., 2012). A single-tailed within-group comparison revealed a required sample size of 15 per group (alpha set to 0.05 and power at 0.95). Therefore, the presented data should be considered as hypothesis generating. Group and time interactions, main effects of time and simple main effects of time were determined by a mixed model ANOVA with repeated measures and Bonferonni correction. Distribution of data was confirmed by Shapiro-Wilks’ test. Homogeneity of variances were assessed using Levenes’ test for homogeneity of variance. Homogeneity of covariance’s was established by Box’s test of equality covariance matrices. Assumptions were unviolated ( *p*>0.05) unless otherwise stated. When Mauchlys’ test of Spericity were violated ( *p*<0.05), a Greenhouse-Geisser estimate was reported. ANOVA are presented as F test [(degrees of freedom, error terms degrees of freedom) F value, *p* value, partial eta-squared (pɳ ^2^)]. *Post-hoc* data are presented as differences in mean ( *M*), 95% CI, *p* value. A chi-squared test for association was conducted between groups and impaired fasting glucose levels at PRE and POST. Data is presented as group mean + standard deviation (SD). An alpha of *p*<0.05 was used to indicate statistical significance.

## Results

### Participants

**Figure 1.**
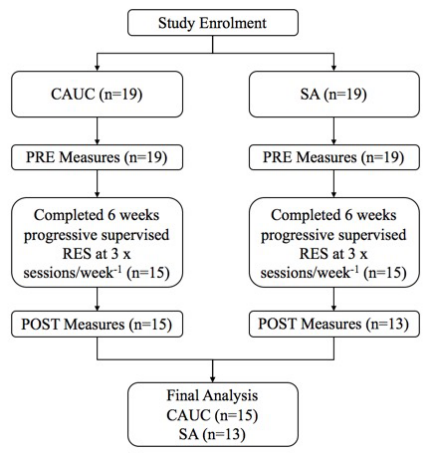
Flow of participants through the study protocol.

Thirty-eight males (n=19 CAUCs, n=19 SAs) were screened and deemed eligible for participation and consequently enrolled. All participants completed PRE measures and initiated supervised RES. Eight participants did not perform POST measures due to non-compliance of the protocol (n=4 CAUCs, n=4 SAs) and two had to withdraw due to personal circumstances (n=2 SAs). The final analysis consisted of 15 CAUC (25.5 ± 4.8 years) and 13 SA (25.4 ± 7.0 years) participants. Because 2 SAs participants withdrew from the study due to personal reasons (bereavement), we elected not to conduct an *intention to treat* analysis (See Figure 1).

### Anthropometrics and systemic blood pressure (mmHg)

No significant group × time interactions were observed following RES for; height, BM, BMI, %BF, FM, FFM, WC, HC, WHR ( *p* > 0.05 for all). No statistically significant main effects of time following RES were identified in any anthropometric measurement ( *p* > 0.05). A simple main effect of time for HC was detected in the CAUC group ( *f*_(1, 24)_ = 4.38, *p* = 0.047, pɳ ^2^ = 0.15) with a significant decrease from PRE-to-POST ( *M* = 3.62 cm, 95% CI: 0.52 – 7.18, *p* = 0.047). No significant main or simple effects of group were observed in any anthropometric measure apart from WHR, where there was a significant main effect of group ( *f*_(1, 24)_ = 5.46, *p* = 0.028, pɳ ^2 ^= 0.19). The CAUC group presented with a significantly higher WHR at PRE ( *M* = 0.52, 95% CI: 0.002 – 0.102, *p* = 0.041) and POST ( *M* = 0.057, 95% CI: 0.006 – 0.107, *p* = 0.029). (See Table 1).

**Table 1.** Descriptive statistics of all variables. Data are presented as group mean ± SD. a - *p*<0.05 from baseline. b - *p*<0.05 between groups at corresponding time point. Abbreviations: BM – body mass (kg), BMI – body mass index (kg/m ^2^), FM – fat mass (kg), FFM – fat free mass (kg), SBP – systolic blood pressure (mmHg), DBP – diastolic blood pressure (mmHg), MAP – mean arterial pressure (mmHg), HOMA-IR – insulin resistance, HDL – high density lipoprotein (mg/dL), LDL – low density lipoprotein (mg/dL), TC – total cholesterol (mg/dL), TRIGS – triglycerides (mg/dL), VEGF – vascular endothelial growth factor (pg/ml), ADMA – asymmetric dimethylarginine (μmol/L), L-ARG – L-arginine (μmol/L), CRP – C-reactive protein (mg/L), CAUC – Caucasian, SA – South Asian, PRE – baseline, POST – following resistance training intervention.

	CAUC		SA		ANOVA Main Effects		
	PRE	POST	PRE	POST	Interaction ( *p*)	Time ( *p*)	Group ( *p*)
**Anthropometrics**							
Height (cm)	176.73 ± 5.98	176.70 ± 4.97	179.69 ± 7.80	179.88 ± 8.27	0.794	0.854	0.237
BM (kg)	81.70 ± 19.70	82.40 ± 19.85	81.96 ± 18.57	83.03 ± 16.62	0.817	0.28	0.951
BMI (kg/m ^2^)	25.97 ± 5.42	26.26 ± 5.71	25.16 ± 4.19	25.46 ± 3.54	0.976	0.220	0.664
Body fat (%)	21.65 ± 1.85	22.01 ± 2.11	21.37 ± 2.01	21.66 ± 2.36	0.917	0.42	0.914
FM (kg)	19.26 ± 2.63	19.74 ± 2.88	18.71 ± 2.94	18.93 ± 3.22	0.75	0.408	0.869
FFM (kg)	63.17 ± 9.19	62.66 ± 8.41	65.36 ± 10.02	63.61 ± 8.01	0.461	0.184	0.644
WC (cm)	90.69 ± 14.04	87.84 ± 16.93	85.46 ± 9.59	83.76 ± 9.58	0.641	0.076	0.353
HC (cm)	106.69 ± 9.78	**103.08 ± 13.21** ^a^	107.46 ± 6.84	106.08 ± 7.77	0.370	0.052	0.606
WHR	0.85 ± 0.07	0.85 ± 0.07	0.79 ± 0.06	0.79 ± 0.05	0.739	0.739	0.028
**Blood pressure**							
SBP (mmHg)	129.04 ± 10.96	128.0 ± 8.88	127.53 ± 10.82	128.77 ± 7.62	0.572	0.961	0.909
DBP (mmHg)	79.0 ± 6.14	**73.61 ± 8.66** ^a^	75.62 ± 8.72	78.08 ± 9.58	0.022	0.373	0.852
MAP (mmHg)	92.66 ± 8.23	89.45 ± 8.21	91.19 ± 8.87	93.28 ± 8.72	0.094	0.717	0.687
**Muscular Strength**							
Lower body (kg)	73.66 ± 16.65	**135.0 ± 27.71** ^a^	68.84 ± 16.47	**111.53 ± 16.88** ^a, b^	0.002	<0.001	0.06
Upper body (kg)	51.66 ± 16.97	**65.33 ± 15.05** ^a^	53.84 ± 15.43	**65.76 ± 14.26** ^a^	0.476	<0.001	0.822
**Blood Biochemistry**							
Glucose (mmol/L)	6.05 ± 1.13	**5.28 ± 0.93** ^a^	6.02 ± 0.63	**5.22 ± 1.14** ^a^	0.96	0.006	0.912
Insulin (μU/ml)	2.83 ± 1.98	3.06 ± 1.98	3.94 ± 2.07	3.50 ± 2.34	0.549	0.849	0.383
HOMA-IR	0.69 ± 0.42	0.60 ± 0.42	1.20 ± 0.70	1.16 ± 0.78	0.826	0.614	0.067
HDL (mg/dL)	35.66 ± 11.93	**46.00 ± 11.21** ^a^	33.50 ± 6.28	**42.66 ± 10.48** ^a^	0.744	<0.001	0.551
LDL (mg/dL)	137.01 ± 40.72	**89.17 ± 21.45** ^a^	138.54 ± 37.22	**81.16 ± 16.86** ^a^	0.572	<0.001	0.793
TC (mg/dL)	189.91 ± 45.01	**147.82 ± 22.48** ^a^	231.88 ± 88.96	**170.90 ± 56.59** ^a^	0.388	<0.001	0.145
TRIGS (mg/dL)	93.27 ± 36.66	87.20 ± 34.39	84.35 ± 42.32	104.22 ± 72.75	0.156	0.442	0.826
VEGF (pg/ml)	36.86 ± 26.28	**70.39 ± 49.91** ^a^	44.86 ± 27.21	**74.65 ± 29.64** ^a^	0.836	0.003	0.687
ADMA (μmol/L)	0.38 ± 0.09	0.38 ± 0.43	0.32 ± 0.12	0.34 ± 0.07	0.667	0.558	0.314
L-ARG (μmol/L)	62.51 ± 10.60	66.49 ± 14.29	71.43 ± 33.90	62.44 ± 12.10	0.341	0.707	0.805
CRP (mg/L)	5.40 ± 2.69	5.89 ± 3.26	5.96 ± 3.58	**9.50 ± 2.64** ^a, b^	0.153	0.065	0.093

No group × time interaction ( *f*_(1, 25)_ = 0.327, *p* = 0.572, pɳ ^2^ = 0.01), main effects of time ( *f*_(1, 25) _= 0.002, *p* = 0.96, pɳ ^2^ = 0.01) or main effects of group were observed ( *f* = _(1, 25)_ = 0.01, *p* = 0.909, pɳ ^2^ = 0.001) in SBP following RES. SBP did not change within the CAUC ( *M* = 1.04 mmHg, 95% CI: -4.63 – 6.69, *p* = 0.710) or SA groups ( *M* = 1.23mmHg, 95% CI: -4.64 – 7.11, *p* = 0.670) following RES and both groups were similar at PRE ( *M* = 1.49 mmHg, 95% CI: -5.15 – 7.14, *p* = 0.724) and POST ( *M* = 0.76 mmHg, 95% CI: -5.81 – 7.35, *p* = 0.812) RES. There was a group × time interaction in DBP following RES ( *f*_(1, 25)_ = 5.95, *p* = 0.022, pɳ ^2^ = 0.20). No main effects of time ( *f*_(1, 25)_ = 0.83, *p* = 0.373, pɳ ^2 ^= 0.03), or main effects of group ( *f*_(1, 25)_ = 0.04, *p* = 0.85, pɳ ^2 ^= 0.001) were evident in DBP following RES. The CAUC group reduced DBP ( *M* = 5.39 mmHg, 95% CI: 0.69 – 10.08 mmHg, *p* = 0.026), with no difference in the SA group ( *M* = 2.46 mmHg, 95% CI: -2.23 – 7.16 mmHg, *p* = 0.290). Both groups were similar at PRE ( *M* = 3.39 mmHg, 95% CI: -2.72 – 9.49 mmHg, *p* = 0.264) and POST ( *M* = 4.46 mmHg, 95% CI: -2.93 – 11.85 mmHg, *p* = 0.225).

A trend was seen for a group × time interaction ( *f*_(1, 25)_ = 3.03, *p* = 0.094, pɳ ^2^ = 0.11) in MAP following RES. No main effects of time ( *f*_(1, 25)_ = 0.14, *p* = 0.72, pɳ ^2^ = 0.01) or main effects of group ( *f*_(1, 25)_ = 0.17, *p* = 0.687, pɳ ^2^ = 0.01) was seen in MAP following RES. No change was seen in the CAUC ( *M* = 3.21 mmHg, 95% CI: 1.14 – 7.57 mmHg, *p* = 0.141) or SA ( *M* = 2.09 mmHg, 95% CI: 1.43 – 6.61 mmHg, *p* = 0.349) group and both groups were similar at PRE ( *M* = 1.47 mmHg, 95% CI: -5.33 – 8.27 mmHg, *p* = 0.660) and POST ( *M* = 3.84 mmHg, 95% CI: 2.88 – 10.55 mmHg, *p* = 0.250).

### Blood biochemistry

**Figure 2.**
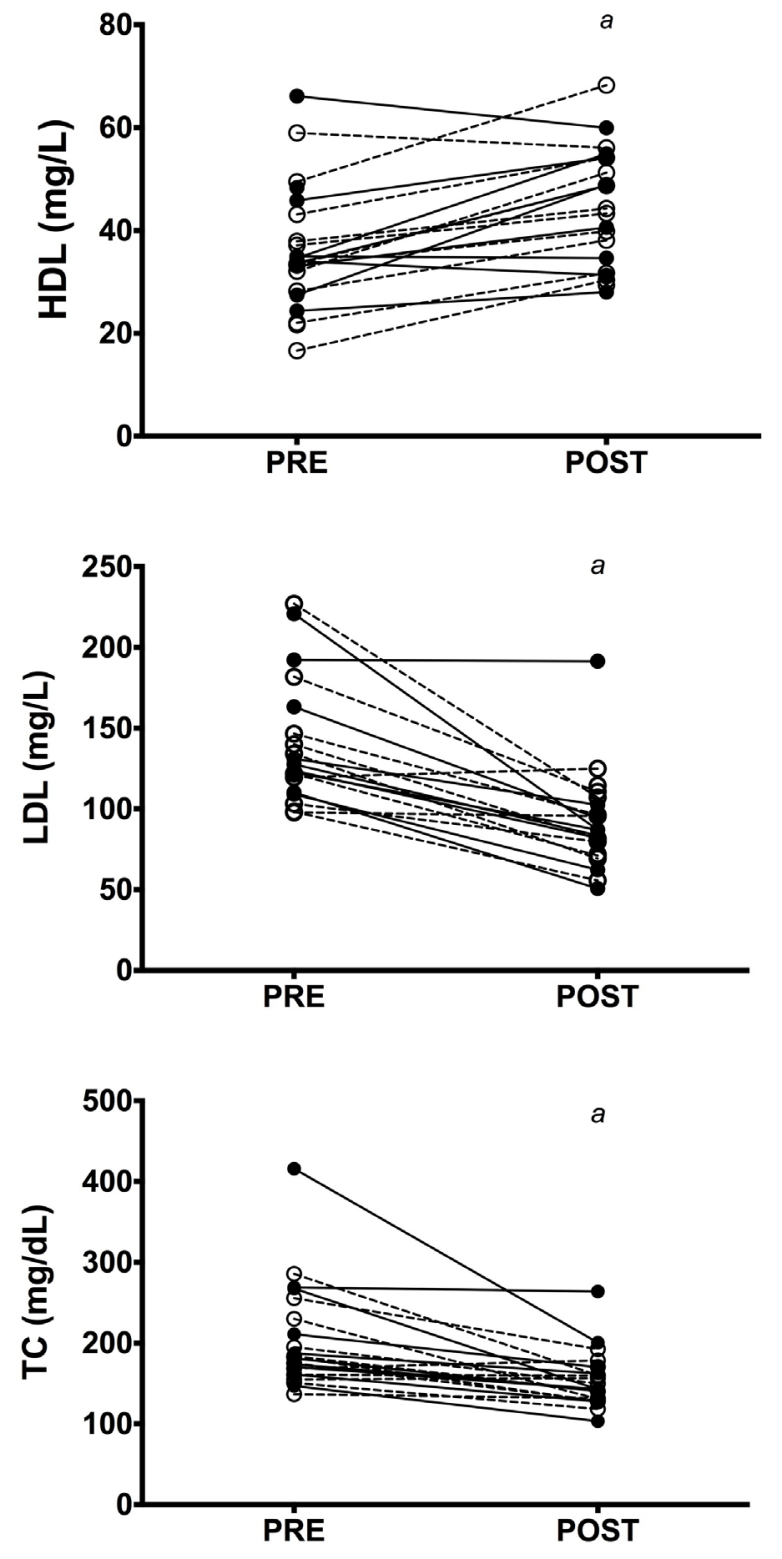
Lipid response to resistance exercise. Both groups presented favourable lipid responses following resistance exercise. *a* – *p*<0.05 from baseline. HDL – high density lipoprotein (mg/dL), LDL – low density lipoprotein (mg/dL), TC – total cholesterol (mg/dL).

**Figure 3.**
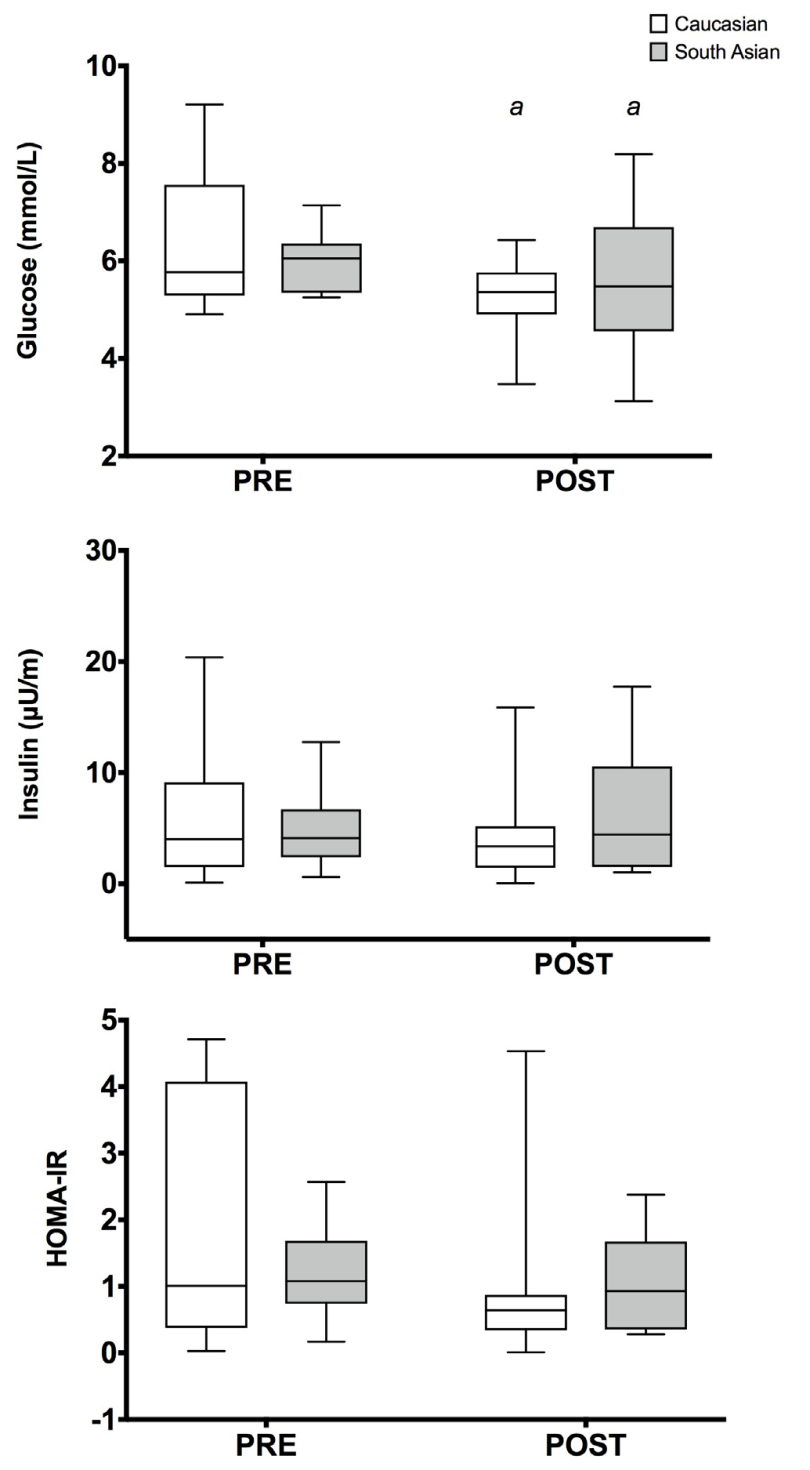
Metabolic response to resistance exercise. Glucose concentrations were significantly lower in both groups following resistance exercise. *a* – *p*<0.05 from baseline. HOMA-IR – insulin resistance.

No group × time interactions were observed in any variable following RES ( *p* > 0.05 for all comparisons). There was a main effect of time in glucose ( *f*_(1, 16)_ = 10.16, *p* = 0.006, pɳ ^2^ = 0.39), TC ( *f*_(1, 21)_ = 23.12, *p* = <0.001, pɳ ^2^ = 0.52), HDL ( *f*_(1, 17)_ = 30.42, *p* = <0.001, pɳ ^2^ = 0.64), LDL ( *f*_(1, 16)_ = 40.39, *p* = <0.001, pɳ ^2^ = 0.72) and VEGF ( *f*_(1, 14)_ = 12.74, *p* = 0.003, pɳ ^2^ = 0.48) following RES. Amongst the CAUC group, there were significant reductions in fasting blood glucose ( *M* = 0.77 mmol/L, 95% CI: 0.03 – 1.51mmol/L, *p* = 0.041), TC ( *M* = 42.09 mg/dL, 95% CI: 11.26 – 72.92mg/dL, *p* = 0.010) and LDL ( *M* = 47.84 mg/dL, 95% CI: 24.44 – 71.23mg/dL, *p* = 0.001), improvements in HDL ( *M* = 10.335 mg/dL, 95% CI: 5.49 – 15.17 mg/dL, *p* = <0.001) and VEGF ( *M* = 33.51 pg/ml, 95% CI: 6.62 – 60.41mg/dL, *p* = 0.018) concentrations (see Figure 2). SA participants enjoyed comparable improvements in fasting blood glucose ( *M* = 0.79 mmol/L, 95% CI: 0.06 – 1.53 mmol/L, *p* = 0.036) TC ( *M* = 60.98 mg/dL, 95% CI: 28.77 – 93.18 mg/dL, *p* = 0.001) and LDL ( *M* = 57.38 mg/dL, 95% CI: 31.22 – 83.53 mg/dL, *p* = <0.001) and increases in HDL ( *M* = 9.16 mg/dL, 95% CI: 3.49 – 14.83 mg/dL, *p* = 0.003), VEGF ( *M* = 29.77 pg/ml, 95% CI: 2.88 – 56.68 pg/ml, *p* = 0.032) and CRP ( *M* = 3.54 mg/L, 95% CI: 0.07 – 7.01 mg/L, *p* = 0.046). Figure 3 shows the metabolic response of both groups.

No main effects of group were seen in any biochemical marker ( *p* > 0.05 for all). A trend was observed for a main effect of group on IR ( *f*_(1, 15)_ = 3.91, *p* = 0.067, pɳ ^2^= 0.207) and CRP ( *f*_(1, 15)_ = 3.22, *p* = 0.093, pɳ ^2 ^= 0.177). Both groups were similar at PRE for glucose ( *M* = 0.03 mmol/L, 95% CI: -0.88 – 0.95 mmol/L, *p* = 0.943), insulin ( *M* = 1.11 U/ml, 95% CI: -1.01 – 3.23 U/ml, *p* = 0.281), TC ( *M* = 41.97 mg/dL, 95% CI: -18.38 – 102.32 U/ml, *p* = 0.163), HDL ( *M* = 2.17 mg/dL, 95% CI: -7.64 – 11.96 mg/dL, *p* = 0.647), LDL ( *M* = 1.53 mg/dL, 95% CI: -37.92 – 40.97 mg/dL, *p* = 0.936), TRIGS ( *M* = 8.92 mg/dL, 95% CI: -25.34 – 43.18mg/dL, *p* = 0.594), VEGF ( *M* = 7.99 pg/ml, 95% CI: -20.69 – 36.68 pg/ml, *p* = 0.560), L-ARG ( *M* = 8.92 μmol/L, 95% CI: -23.39 – 41.24 μmol/L, *p* = 0.552), ADMA ( *M* = 0.06 μmol/L, 95% CI: -0.09 – 0.20 μmol/L, *p* = 0.376) and CRP ( *M* = 0.57 mg/L, 95% CI: -2.69 – 3.83 mg/L, *p* = 0.717). A trend was seen at PRE for IR between groups ( *M* = 0.51, 95% CI: -0.10-1.12, *p* = 0.096).

**Figure 4.**
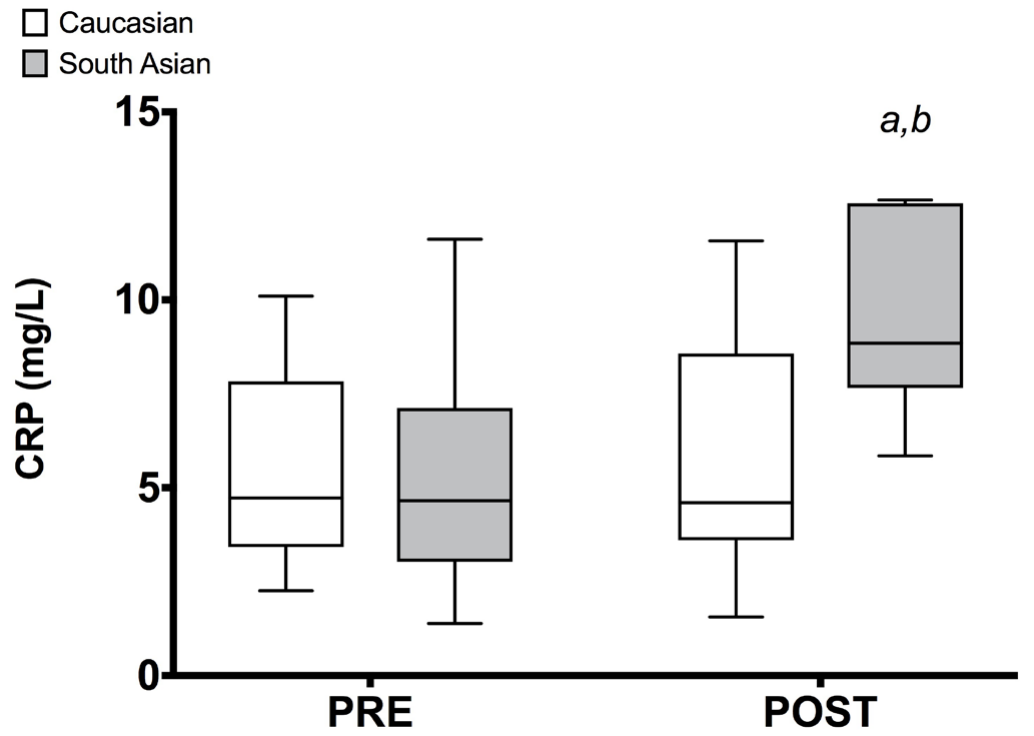
C-reactive protein (CRP) changes following resistance exercise. C-reactive protein concentrations increased in South Asians following resistance exercise, with no change observed within the Caucasian group. *a – p*<0.05 from baseline, *b – p*<0.05 between groups.

**Figure 5.**
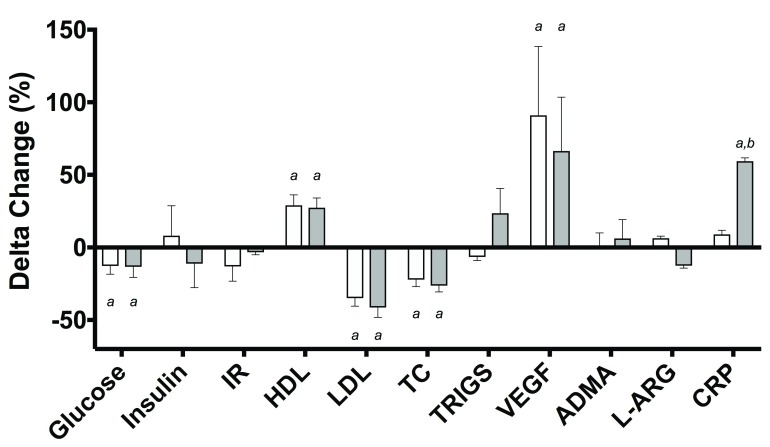
Delta percentage changes of all biochemical measures. Data are presented as means ± SD. *a* - *p*<0.05 within groups. *b* - *p*<0.05 between groups. Abbreviations: IR – insulin resistance, HDL – high density lipoprotein (mg/dL), LDL – low density lipoprotein (mg/dL), TC – total cholesterol (mg/dL), TRIGS – triglycerides (mg/dL), VEGF – vascular endothelial growth factor (pg/ml), ADMA – asymmetric dimethylarginine (μmol/L), L-ARG – L-arginine (μmol/L), CRP – C-reactive protein (mg/L).

At POST, both groups were comparable for glucose ( *M* = 0.06 mmol/L, 95% CI: -0.98 – 1.10 mmol/L, *p* = 0.910), insulin ( *M* = 0.45 μ/ml, 95% CI: -1.79 – 2.69 μ/ml, *p* = 0.676), TC ( *M* = 23.09 mg/dL, 95% CI: -13.64 – 59.81 mg/dL, *p* = 0.205), HDL ( *M* = 3.34 mg/dL, 95% CI: -7.36 – 14.05 mg/dL, *p* = 0.519), LDL ( *M* = 8.01 mg/dL, 95% CI: -11.67 – 27.69 mg/dL, *p* = 0.401), TRIGS ( *M* = 17.02 mg/dL, 95% CI: -31.62 – 65.66 mg/dL, *p* = 0.475), VEGF ( *M* = 4.28 pg/ml, 95% CI: -39.76 – 48.28 pg/ml, *p* = 0.839), L-ARG ( *M* = 4.05 μmol/L, 95% CI: -12.96 – 21.07 μmol/L , *p* = 0.608), ADMA ( *M* = 0.04 μmol/L, 95% CI: -0.04 – 0.12 μmol/L, *p* = 0.317) with a trend evident for IR ( *M* = 0.55, 95% CI: -0.10 – 1.22, *p* = 0.090). The SA group presented higher CRP levels at POST ( *M* = 3.61 mg/L, 95% CI: 0.29 – 6.93 mg/L, *p* = 0.035, see Figure 4). Figure 5 illustrates the percentage change of biomarkers in both groups.

### Muscular strength

A significant group × time interaction was evident for lower body strength ( *f*_(1,26)_ = 11.23, *p* = 0.002, pɳ ^2^ = 0.30). A main effect of time was also observed ( *f*_(1,26)_ = 349.64, *p* < 0.001, pɳ ^2^ = 0.93). The CAUC ( *M* = 61.33 Kg, 95% CI: 53.54 – 69.13 Kg, *p* < 0.001) and SA ( *M* = 42.69 Kg, 95% CI: 34.32 – 51.06 Kg, *p* < 0.001) groups significantly improved lower body strength. Both groups presented similar lower body strength at PRE ( *M* = 4.82 Kg, 95% CI: -8.21 – 17.85 Kg, *p* = 0.454) however, the CAUC group demonstrated higher strength at POST than the SA group ( *M* = 23.46 Kg, 95% CI: 5.28 – 41.65 Kg, *p* = 0.013).

No group × time interaction was observed for upper body strength ( *f*_(1,26)_ = 0.52, p = 0.476, pɳ ^2^ = 0.02). A main effect of time was seen ( *f*_(1,26)_ = 112.89, *p* < 0.001, pɳ ^2^ = 0.81). The CAUC ( *M* = 13.67 Kg, 95% CI: 10.29 – 17.04 Kg, *p* < 0.001) and SA ( *M* = 11.92 Kg, 95% CI: 8.30 – 15.55 Kg, *p* < 0.001) both improved upper body strength. Both groups were similar in upper body strength at PRE ( *M* = 2.18 Kg, 95% CI: -10.50 – 14.86 Kg, *p* = 0.727) and POST ( *M* = 0.44 Kg, 95% CI: -11.01 – 11.88 Kg, *p* = 0.94).

## Discussion

The main findings of the current study demonstrate comparable responses of biomarkers of cardio-metabolic health between CAUC and SA males in response to short-term progressive RES. These responses were evident despite significant differences in muscular strength adaptation. Both groups significantly improved lipid profiles, fasting glucose and VEGF following RES. Although, no difference was observed in fasting TRIGS, insulin, L-ARG, ADMA, or IR following RES in any group, with no apparent discrepancy between groups. CRP increased in the SA group following RES with no change evident in CAUCs.

Previous findings have reported that increasing physical activity levels is beneficial for cardio-metabolic risk in SAs ( Dhawan & Bray, 1997; Eriksen *et al*., 2015; Mathews *et al*., 2007) ( Dhawan & Bray, 1997; Eriksen *et al*., 2015; Mathews *et al*., 2007). Dhawan & Bray (1997) showed marked differences in physical activity levels between CAUCs and SAs, however increased physical activity was associated with reductions in insulin, BMI, TRIGS, and blood pressure in both groups. Similarly, Mathews *et al*. (2007) reported improvements in body composition, blood pressure, and lipid profiles following the Khush Dil initiative which involved participation in physical activity and nutritional workshops. Data from the SABRE study reported positive associations between physical activity and markers of cardio-metabolic health in SAs ( Eriksen *et al*., 2015). However, conflicting data suggest a blunted response to physical activity in the SAs cohort.

Data from the Indian Diabetes Prevention Programme ( Ramachandran *et al*., 2006) show no change in body mass following diet and physical activity advice in native Indians, although the intervention group did have a significantly lower incidence of diabetes development at year 3 than the controls. The lack of change in physiological markers are in agreement with later data that show no positive associations with increasing levels of physical activity and body composition in SAs ( Yates *et al*., 2010). These data are supported by a more recent intervention which resulted in mixed body composition responses in SAs ( Bhopal *et al*., 2014).

These studies not only have a key limitation of self-reported measures of physical activity, but also provide difficulty in establishing a dose-response relationship between racial groups to an exercise stimulus. The current study, to our knowledge, is the first to demonstrate a comparable cardio-metabolic response to RES between CAUC and SA males. These data provide evidence that RES is an effective method to improve cardio-metabolic health in the high-risk SA population, with no apparent discrepancy in response when compared with CAUCs. Considering previous investigations have reported a blunted response to aerobic exercise in SAs, the current data provides intriguing evidence that RES interventions may prove superior to aerobic exercise to improve cardio-metabolic health in young SAs. Further research is warranted to determine this theory.

The limited exercise response suggested by the results from previous associations of physical activity and cardio-metabolic health in SAs has initiated several comparative studies. Hall *et al*. (2010) demonstrated that SAs have a reduced capacity to oxidise fat during submaximal aerobic exercise in comparison with age and body composition matched CAUCs. These data suggest the SAs may need to participate in longer durations or higher exercise intensities to elicit the same physiological benefit from exercise. More recent evidence showing a blunted response in SAs from the same research group used age-adjusted regression models to determine the association between moderate intensity physical activity and cardio-metabolic risk ( Iliodromiti *et al*., 2016). This study reports that middle-aged SA men and women need to participate in 232 min/week ^-1^ to elicit comparable cardio-metabolic benefits as CAUCs who perform the currently recommended 150 min/week ^-1^. Taken these data together, they suggest a blunted response to exercise in the SA cohort. However, the current investigation demonstrates a comparable cardio-metabolic response to RES between young CAUC and SA males. Further study is warranted to establish if the ability to utilise metabolic substrates during RES is varied between racial groups. The lack of RES studies within the SA population provides difficulty to establish a comparison of existing data which potentially can contribute to our understanding of optimal RES prescription. Nevertheless, the current study demonstrates that progressive RES is effective at improving cardio-metabolic health in young CAUCs and SAs. These data also demonstrate the importance of targeting younger SA individuals to reduce the risk of developing cardio-metabolic diseases that older generations have suffered.

CRP has been regarded as an emerging risk factor for the development of cardiovascular diseases that involve systemic inflammation ( Singh *et al*., 2008). It has been reported that SA males have a 17% higher concentration of CRP than matched CAUC males, with differences seen in individuals as young as 10 years old ( Chambers *et al*., 2001; Cook *et al*., 2000). The current literature concerning the effect of RES on circulating CRP levels is mixed. RES has been shown to reduce CRP levels in African American males with no difference observed in White Americans ( Heffernan *et al*., 2009). These results may have been a consequence of differential baseline values as the African American group presented higher levels of CRP. Additional work has also reported no change in CRP levels following 10 weeks of RES in individuals with metabolic risk factors ( Levinger *et al*., 2009). The current study is in agreement with previous research as no changes were seen in the CAUC group. The lack of change in CRP levels following RES within the CAUC group may have been a consequence of no statistical change in body composition as there is a correlation between CRP levels and BMI ( Vlachopoulos *et al*., 2015). However, a significant increase was seen in the SA group. This may suggest that adequate recovery from a training bout may take longer in the SA group. Previous studies have reported abnormal heart rate recovery from a graded exercise test to be positively associated with CRP levels, independent of physical fitness and disease status ( Jae *et al*., 2007). Although the current data cannot provide evidence on recovery rates, an abnormal recovery from an exercise bout may be present in the SA population. This requires further exploration.

The current findings may have important clinical implications. According to current guidelines ( American Diabetes Association, 2007), both groups demonstrated impaired fasting glucose at baseline. RES resulted in a significant improvement in both groups to normal fasting glucose levels, suggesting that RES protocols similar to that employed in the current study is sufficient to reduce the risk of type 2 diabetes development. The significant improvement in lipid profiles also demonstrates the efficacy of RES in improving cardio-metabolic health in young CAUC and SA males. Both groups demonstrated significant improvements in TC, LDL and HDL concentrations, which may translate to a significant reduction in cardiovascular risk. This is of importance as our previous work has demonstrated differing rates of adaptation to exercise training between CAUCs and SAs ( Knox *et al*., 2017), which may imply differing rate of adaptation in other physiological markers. However, the current study has demonstrated that this is not evident in biomarkers of cardio-metabolic health.

This study does not come without several limitations. The small sample size of both racial groups may not provide sufficient statistical power to determine differences in the biomarkers that lacked disparity between groups in response to RES. Nevertheless, the current data would suggest that the biomarkers associated with cardio-metabolic health would respond similarly between CAUCs and SAs in response to RES. The small sample size also makes these data difficult to generalise to whole populations. Additionally, as previous literature using self-reported physical activity levels has suggested a blunted response to exercise in the SA population, a larger sample size that includes participants of similar age to existing literature would be necessary for validation. A larger profile of biomarkers would have also been necessary. There is strong evidence of emerging risk factors such as plasminogen activator inhibitor-1, lipoprotein A, homocysteine, tumor necrosis factor A and several adhesion molecules that are all linked with the development of cardiovascular disease. Interestingly, these compounds seem to be more prevalent in SA individuals when compared to CAUCs ( Gupta *et al*., 2006). Inclusion of these measures would have given greater detail in the efficacy of RES in the prevention of cardiovascular risk between CAUCs and SAs. Future research concerning the SA population and RES should consider these emerging risk factors to develop the limited knowledge of exercise response within this population.

In conclusion, supervised progressive RES is effective at improving biomarkers of cardio-metabolic health within young CAUC and SA males, with no apparent discrepancy in response to exercise training. SAs significantly increased CRP levels which may indicate a requirement of a longer recovery following exercise. However, this theory has yet to be confirmed. These data provide an evidence base for interventions to target the younger generation of SAs which may assist in the reduction of future cardio-metabolic diseases that has been prevalent in the SA population for decades.

## Data availability

Underlying data for this study is available from *figshare*: Dataset 1. Short-Term Resistance Exercise and Cardiometabolic Health in Caucasian and South Asian Males, https://doi.org/10.6084/m9.figshare.6741731.v1 ( Knox *et al*., 2018)

Data available under CC0 1.0 Universal licence
