# Supplementary material for: Caucasian and south Asian men show equivalent improvements in surrogate biomarkers of cardiovascular and metabolic health following 6-weeks of supervised resistance training
Source: F1000Res. 2019 Mar 5;7:1334. Originally published 2018 Aug 24. [Version 2] doi: 10.12688/f1000research.15376.2 (PMC6419981; doi:10.12688/f1000research.15376.2)
Supplement: Peer review history

# Peer review: Reviewer response for version 2

**DOI:** 10.5256/f1000research.20179.r45264

**Published:** 2019-03-13

**Authors:** Eliza Prodel

**Affiliations:** 1 Laboratory of Exercise Sciences, Department of Physiology and Pharmacology, Fluminense Federal University, Niteroi, Brazil

The authors have satisfactorily improved the manuscript. Therefore, I now recommend the publication.

I have read this submission. I believe that I have an appropriate level of expertise to confirm that it is of an acceptable scientific standard.

# Peer review: Reviewer response for version 1

**DOI:** 10.5256/f1000research.16755.r41730

**Published:** 2018-12-11

**Authors:** Majid Ghayour-Mobarhan

**Affiliations:** 1 Cardiovascular Research Center, Mashhad University of Medical Science, Mashhad, Iran

The manuscript by Knox et al., tries to compare the response in biomarkers of cardio-metabolic health in Caucasians and South Asians in response to resistance exercise. The study appears to have been done in a careful and competent manner. I have few comments that require further clarification.

 Method part: Why were samples selected from male participants in the study? Please explain more in this regard. While more details of method will provide to allow replication by others.

 Results and discussion parts: Also please include other anthropometric indices as CVD risk factors such as the waist circumference-to-hip circumference ratio, ect in the text, if it is possible because it has been proposed to assess central obesity and as measures for cardio-metabolic disorders and events.

 Therefore, insert and explain the associations them in the result and discussion sections.

I have read this submission. I believe that I have an appropriate level of expertise to confirm that it is of an acceptable scientific standard.

# Peer review: Reviewer response for version 1

**DOI:** 10.5256/f1000research.16755.r38063

**Published:** 2018-09-21

**Authors:** Eliza Prodel

**Affiliations:** 1 Laboratory of Exercise Sciences, Department of Physiology and Pharmacology, Fluminense Federal University, Niteroi, Brazil

In the paper entitled “Caucasian and south Asian men show equivalent improvements in surrogate biomarkers of cardiovascular and metabolic health following 6-weeks of supervised resistance training” you had the aim to investigate the cardio-metabolic response to resistance exercise training in South Asian and Caucasian population. The work is well written, and presents very interesting data, however a have a few issues to clarify.

 Major

 The whole introduction is based on the fact that SA’s are cardio-metabolic different from CAUCC’s, so why you hypothesized no differences would be found in the results?

 Why only male participants were enrolled in the study? You would have more complete results and an avenue to discuss if women were tested.

 Data of muscular strength gain could be more clear in a table or graph.

 It is not mandatory, however I feel that the authors could explore more about cardiovascular data and their relationship with the metabolic data. For example, changing in rest and recovery heart rate and heart rate variability with the RES training.

 Methods

 Participants section:

1. Performance measurements (upper and lower body) should be in a specific section, and would be appropriate to provide more detailed information.
2. “A single-tailed within-group comparison revealed a required sample size of 15 per group (alpha set to 0.05 and power at 0.95). Therefore, the presented data should be considered as hypothesis generating.” Should be in statistical analysis section.

 

 Minor

 Page 5: “Neither was there any change within in the CAUC”. Small writing mistake.

 Page 8: “however, the CAUC group showed demonstrated higher strength at POST than the SA group”. Small writing mistake.

I have read this submission. I believe that I have an appropriate level of expertise to confirm that it is of an acceptable scientific standard, however I have significant reservations, as outlined above.

# Response

**Published:** 2019-02-26

**Authors:** Allan Knox

**Affiliations:** California Lutheran University, USA

The authors thank you for taking the time to review our manuscript. We have considered all comments and provided our responses below;

 Comment: The whole introduction is based on the fact that SA’s are cardio-metabolic different from CAUCC’s, so why you hypothesized no differences would be found in the results?

 Response: We have clarified our hypothesis to reflect the background information that is reported in the introduction of the manuscript.

 Comment: Why only male participants were enrolled in the study? You would have more complete results and an avenue to discuss if women were tested.

 Response: There is paucity of experimental exercise research in south Asian males and none in south Asian females. We sought to focus on the male population to control for the possibility of an effect of sex in responses to resistance exercise training. We agree that more data is required on exercise responses in south Asian females, something that we wish to explore in a more comprehensive sample in future experimental work.

 Comment: Data of muscular strength gain could be more clear in a table or graph.

 Response: We thank you and appreciate your comment regarding the request for a graph illustrating muscular strength gains. We feel it is unfeasible to provide additional graphs for strength gains due to the number of muscle groups trained. All data regarding strength gains is presented in tables. In addition, these tables are more informatory presented as means and standard deviations due to the differences in strength response.

 Comment: It is not mandatory, however I feel that the authors could explore more about cardiovascular data and their relationship with the metabolic data. For example, changing in rest and recovery heart rate and heart rate variability with the RES training.

 Response: We fully appreciate this comment and are considering exploring these data in more detail.

 Comment: Performance measurements (upper and lower body) should be in a specific section, and would be appropriate to provide more detailed information.

 Response: We are constrained by the word count available to provide additional information regarding the performance measures. We have referenced previous research that has full details of all procedures.

 Comment: “A single-tailed within-group comparison revealed a required sample size of 15 per group (alpha set to 0.05 and power at 0.95). Therefore, the presented data should be considered as hypothesis generating.” Should be in statistical analysis section.

 Response: This has now been moved to the statistical analysis section.

 Comment: Page 5: “Neither was there any change within in the CAUC”. Small writing mistake

 Response: Amended in manuscript.

 Page 8: “however, the CAUC group showed demonstrated higher strength at POST than the SA group”. Small writing mistake.

 Response: Amended in manuscript.
